# A molecular picture of surface interactions of organic compounds on prevalent indoor surfaces: limonene adsorption on SiO_2_†
†Electronic supplementary information (ESI) available: Supplementary information contains a table with mode assignments for limonene adsorbed on SiO_2_, additional information on the surface coverage calculation and two additional figures. The first figure (Fig. S1) is of the H(silica)–C radial distribution functions computed for the sp^3^ and sp^2^ carbon atoms calculated for the full trajectory and structures corresponding to the C* up and C* down conformations. The second figure (Fig. S2) shows time course measurements of limonene concentrations on SiO_2_ for fourteen different limonene pressures. See DOI: 10.1039/c8sc05560b


**DOI:** 10.1039/c8sc05560b

**Published:** 2019-01-09

**Authors:** Yuan Fang, Pascale S. J. Lakey, Saleh Riahi, Andrew T. McDonald, Mona Shrestha, Douglas J. Tobias, Manabu Shiraiwa, Vicki H. Grassian

**Affiliations:** a Department of Chemistry & Biochemistry , University of California , San Diego , La Jolla , 92093 , CA , USA . Email: vhgrassian@ucsd.edu; b Department of Chemistry , University of California , Irvine , 92697 , CA , USA . Email: m.shiraiwa@uci.edu ; Email: dtobias@uci.edu; c Scripps Institution of Oceanography , Department of Nanoengineering , University of California , San Diego , La Jolla , 92093 , CA , USA

## Abstract

Indoor surfaces are often coated with organic compounds yet a molecular understanding of what drives these interactions is poorly understood. Herein, the adsorption and desorption of limonene, an organic compound found in indoor environments, on hydroxylated silica (SiO_2_) surfaces, used to mimic indoor glass surfaces, is investigated by combining vibrational spectroscopy, atomistic computer simulations and kinetic modeling. Infrared spectroscopy shows the interaction involves hydrogen-bonding between limonene and surface O–H groups. Atomistic molecular dynamics (MD) simulations confirm the existence of π-hydrogen bonding interactions, with one or two hydrogen bonds between the silica O–H groups and the carbon–carbon double bonds, roughly one third of the time. The concentration and temperature dependent adsorption/desorption kinetics as measured by infrared spectroscopy were reproduced with a kinetic model, yielding the adsorption enthalpy of ∼55 kJ mol^–1^, which is consistent with the value derived from the MD simulations. Importantly, this integrated experimental, theoretical and kinetic modeling study constitutes a conceptual framework for understanding the interaction of organic compounds with indoor relevant surfaces and thus provides important insights into our understanding of indoor air chemistry and indoor air quality.

## Abstract 2

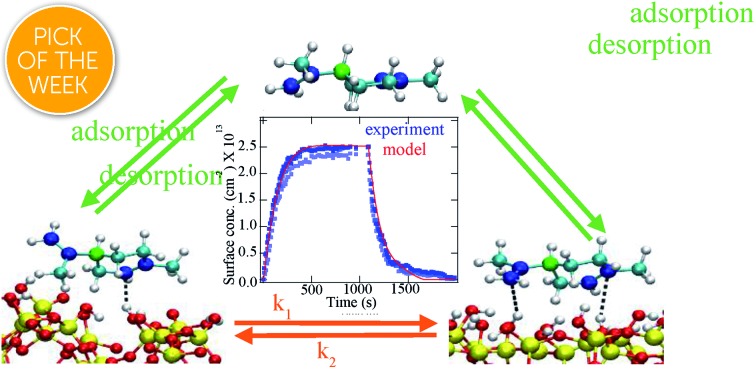
Integration of experiment, theory and modeling to understand the interaction type and kinetics of limonene on silica surfaces.

## Introduction

Organic compounds are highly prevalent in the indoor environment.^1^ Coming from a variety of sources including outdoor exchange, occupants, personal care and cleaning products, furniture, and building materials, these organic compounds can form films on various impermeable indoor surfaces through processes such as adsorption and deposition.^1^,^2^ Organic films have been found to be 5 to 30 nm thick forming at a faster rate initially followed by a slower growth with time.^2^–^6^ Besides simple adsorption, indoor surfaces could also provide substrates for reactions to occur that may lead to the formation of new molecular species that can remain on the surface or desorb into the gas phase depending on the strength of the interaction with the surface and the volatility of the product.^7^,^8^ For example, given the presence of oxidants such as ozone, hydroxyl radicals and nitrogen dioxide, these organic films could also be oxidized to form oxygenated compounds, compounds that are precursors to secondary organic aerosol formation.^9^,^10^ Since carbon dioxide and ammonia are also present indoors (at parts per million levels), with their concentrations varying as occupants change activities, acid–base reactions are potentially important as well for organic films accumulated on indoor surfaces.^11^,^12^ Furthermore, as indoor spaces consist of numerous surfaces such as windows, walls, and floors, resulting in a large surface to volume ratio, interfacial chemistry can significantly impact the indoor air quality and health of occupants.^1^,^7^ However, there remains a lack of knowledge concerning the detailed chemistry and fundamental molecular interactions involving organics that occur at indoor surfaces. Since these processes will impact indoor air quality, coupled to the fact that people spend almost 90% of their time indoors in industrialized nations, it is important to have an understanding of molecular processes in the indoor environment that occur on indoor surfaces.

Among the various surface chemical processes occurring on indoor surfaces, here we have focused our attention on elucidating the fundamental interactions, a detailed molecular picture, and the kinetics of the adsorption/desorption process of a prevalent indoor gas, d-limonene, on hydroxylated SiO_2_, a model for glass surfaces which are prominent in indoors. Limonene, a terpene commonly found in the indoor environment, is an active ingredient in a variety of consumer products such as cleaning products and odorants.^13^,^14^ It has been found to form secondary organic aerosol upon oxidation in the indoor environment.^14^–^17^ The average reported indoor concentration of limonene is 5–15 ppb,^18^ however, the concentration can escalate up to hundreds of ppb following product use.^14^ Furthermore, SiO_2_ was chosen as the model indoor surface in this study since it can represent the chemistry occurring on glass surfaces, which are abundant in the indoor environment. The SiO_2_ sample used in our study is in powder form, having a high surface area, to allow us to obtain detailed information on surface adsorption mechanisms.

To our knowledge, this is the first investigation of the interaction type, strength, and kinetics of limonene adsorption/desorption on SiO_2_ surface by integrating surface adsorption measurements obtained from vibrational spectroscopy with theoretical calculations and kinetic modeling. Using MD simulations, limonene is found to adsorb onto the surface by forming one or two π hydrogen bonds with the hydroxyl groups of SiO_2_. Infrared experiments corroborate the formation of hydrogen bonds upon exposure of limonene to SiO_2_. Furthermore, the kinetic double-layer model for surface chemistry (K2-SURF)^19^ based on parameters validated by MD simulations was able to reproduce experimental kinetic results of limonene adsorption/desorption on SiO_2_. The methods developed, and the kinetic model built here for limonene adsorption on SiO_2_ can be applied to other indoor organic vapors and surfaces, which will eventually lead to important laboratory data needed for modeling indoor air quality. This study represents an important first step in developing a molecular understanding of indoor surface chemistry.

## Experimental procedures

### Transmission FTIR surface spectroscopy

The adsorption of limonene on SiO_2_ surfaces as a function of limonene pressure at 296 ± 1 K, as well as from 298 to 308 K for temperature dependence experiments were studied using transmission Fourier transform infrared (FTIR) spectroscopy coupled with a modified Teflon coated infrared cell.^20^,^21^ In these experiments, approximately 5 mg of SiO_2_ particles (Degussa, BET surface area of 230 m^2^ g^–1^), was pressed onto one half of a tungsten grid held by two Teflon coated jaws in the FTIR cell compartment (177 ± 2 mL). The customized Teflon coated infrared cell is connected to a glass mixing chamber (1329 ± 2 mL) *via* Teflon tubing (75 cm long, with a diameter of 3.3 mm). The sample cell and mixing chamber were then evacuated for 6 hours using a turbo-molecular pump to clean the cell and the sample surface. After evacuation, the sample was exposed to the desired pressures of dry, gaseous limonene for 20 minutes under dry conditions (RH < 1%). The gaseous limonene was produced from (+)-limonene (>99%, Fisher Scientific) by degassing at least three times with consecutive freeze–pump–thaw cycles. Limonene adsorption/desorption at 296 K were studied at a series of 14 equilibrium pressures (6, 7, 8, 9, 10, 12, 13, 14, 16, 18, 21, 22, 23, and 28 mTorr), with 3 replicas carried out at 7, 14, and 22 mTorr. To investigate the effects of temperature, the SiO_2_ sample was held by a custom heated sample holder as described in previous studies.^22^,^23^ Thermocouple wires are welded to the tungsten grid to measure the temperature of the sample and the tungsten grid, with the sample coated on the grid resistively heated by an external heater. With the heated sample holder, the volume of the FTIR cell increased to 327 (±3) mL. The coated surface samples were evacuated for at least 6 hours and pre-heated to the desired temperature before introduction of limonene.

Prior to and after the exposure of limonene, the single-beam spectra of surface- and gas- phases (300 scans) were acquired at 296 K, as well as from 298 K to 308 K for temperature dependence experiments. The following temperatures were employed for these experiments: 298, 300.5, 303, 305.5 and 308 K. A resolution of 4 cm^–1^ was used over the spectral range of 800 to 4000 cm^–1^. As SiO_2_ is opaque below ∼1200 cm^–1^, spectra are shown only above 1200 cm^–1^. During and following exposure to limonene, single-beam spectra (10 scans) of the respective SiO_2_ and gas phase surfaces were automatically acquired using a Macro (OMNIC Macro Basics software) to study the kinetics of limonene adsorption until equilibrium was reached. The IR cell and sample surface were evacuated after adsorption had reached equilibrium. Desorption information was then obtained by acquiring single-beam spectra (10 scans) for 30 minutes. Absorbance spectra of limonene on the SiO_2_ surface are reported as the difference in the SiO_2_ spectra before and following exposure to limonene. Absorption bands attributed to gas phase limonene (measured through the blank half of the tungsten grid) were subtracted from the surface absorbance spectra to obtain the FTIR spectra of the adsorbed particle species loaded on the tungsten grid.

### MD simulations

The amorphous SiO_2_ structure was generated applying an annealing procedure.^24^ Initially, an alpha-quartz supercell composed of 11 × 11 × 8 unit cells was built. To accommodate periodic boundary conditions, bonds were introduced between the atoms located at the borders of the crystal with their bonding partners located at the opposite surface. Upon completion of the annealing process a 24 × 52 × 50 Å^3^ slab was selected from the annealed bulk structure. To ensure that all Si atoms located at the surface in the *X* direction (normal to the SiO_2_ surface) satisfied tetrahedral coordination, a few oxygen atoms were added to the system. In the simulations involving the SiO_2_ slab, a 40 Å thick region of vacuum was added to both sides of the slab in the *X* direction. The hydroxylated SiO_2_ surface was generated by hydrogenating the surface oxygen atoms that had only one Si–O bond, resulting in a silanol surface density of 6.7 nm^–2^.

All of the MD simulations were performed using the LAMPPS package.^25^ The equations of motion were integrated using the velocity-Verlet algorithm with a 1 fs time step. Electrostatic interactions were evaluated with the particle–particle particle–mesh solver^26^ with a 14 Å cutoff distance for the short-ranged nonbonded interactions. The simulation temperature was maintained at 295 K using the Nosè–Hoover thermostat with a relaxation time of 100 fs. CHARMM-compatible bonded and nonbonded force field parameters optimized for the SiO_2_ (ref. 26) were employed, and the CHARMM CGenFF force field^27^ parameters were used for limonene. Subsequently, a d-limonene molecule was introduced in the vacuum region adjacent to the slab and a 0.5 μs MD trajectory was generated at constant volume and a constant temperature of 295 K.

Umbrella sampling^28^ was employed to calculate the potential of mean force (PMF) or free energy profile for the desorption of limonene from the SiO_2_ surface. The distance of the center of mass of limonene from the surfaces was chosen as the reaction coordinate, and the desorption process was divided into 31 windows at 0.5 Å increments. A harmonic restraining potential with a force constant of 20.9 kJ mol^–1^ Å^–2^ was applied in each window. The free energy profile was generated from 10 ns long biased trajectories for each PMF window using the WHAM scheme.^29^ The limonene desorption enthalpy was estimated by energy minimization as a function of the separation between the limonene center of mass and SiO_2_ surface. This procedure was repeated for 100 different initial structures extracted from the 0.5 μs MD simulation.

During the MD simulation we observed predominantly two configurations of the limonene molecule on the silica surface, which we refer to as C* up and C* down based on the proximity of the chiral carbon atom to the surface. The activation energies for the transitions from the C* up to the C* down and the C* down to C* up configurations were estimated from the relative energies of the C* up, C* down, and vertical orientations of the limonene ring with respect to SiO_2_ surface. The energies were calculated at the M06-2X/6-311++G(d,p) level^30^ using structures of SiO_2_ and limonene optimized at the M06-2X/6-311G(d) level. The energies were corrected for basis set superposition error.^31^ The initial structures for these calculations were obtained from the force field-based MD simulation. The size of the SiO_2_ cluster used in the electronic structure calculations, 112 atoms, was chosen such that it can fully contain the different orientations of the limonene molecule. All of the electronic energy calculations were performed using the GAUSSIAN16 package.^32^

### K2-SURF model description and parameters

The K2-SURF model was used to reproduce experimental measurements of adsorbed limonene concentrations on a SiO_2_ surface as a function of pressure and temperature. Adsorption and desorption of limonene to and from the SiO_2_ surface were included in the model. Adsorbed limonene could be either bound to the surface in the C* down or C* up configuration, and the interconversion between these configurations was explicitly treated in the model. The first-order desorption rate coefficient was assumed to follow Arrhenius kinetics. The gas-phase limonene pressure in the reaction cell as a function of time was constrained to experimental measurements. It was also assumed that gas-phase diffusion into the SiO_2_ matrix, which was ∼150 μm thick, was fast and therefore did not affect the measured adsorption and desorption kinetics.

## Results and discussion

### Limonene adsorption on hydroxylated SiO_2_: surface vibrational spectroscopy

**Fig. 1:**
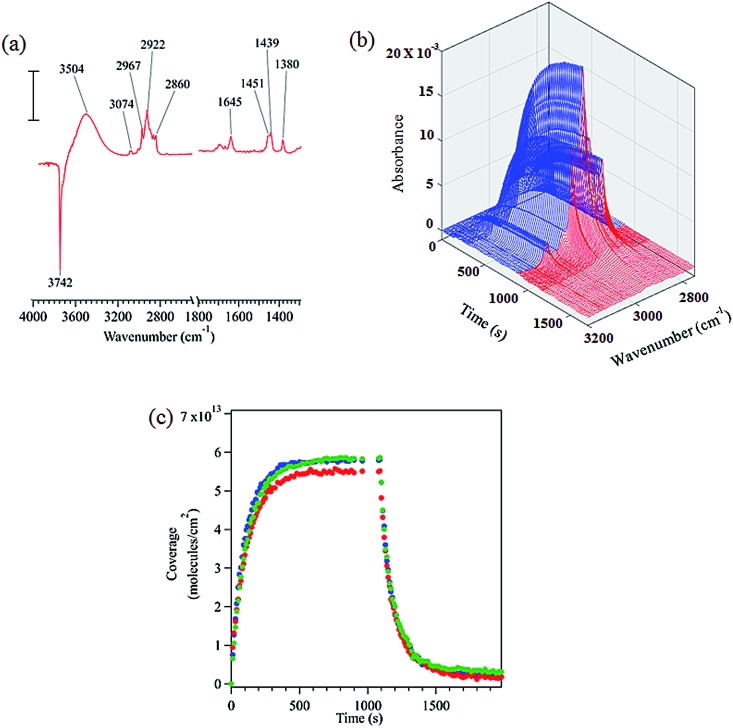
(a) FTIR spectrum of limonene adsorbed on SiO_2_ surface at 22 mTorr equilibrium pressure (scale bar = 0.01 absorbance units). (b) Absorbance spectra in the C–H stretching region as a function of time during adsorption (blue) and desorption (red). (c) Temporal evolution of the adsorption and desorption of limonene on SiO_2_ (triplicate experiments are shown).

FTIR spectroscopy shows that limonene adsorbs to the hydroxylated SiO_2_ surface *via* hydrogen bonding as shown by the loss of isolated surface hydroxyl groups at 3742 cm^–1^ and the redshifted broad peak centered at 3504 cm^–1^ (see Fig. 1a). Based on similar results, previous infrared studies have also reported the presence of hydrogen bonding between other molecules and the SiO_2_ surface.^20^,^33^,^34^ A detailed peak assignment for limonene adsorbed on hydroxylated SiO_2_ is provided in the ESI (Table S1†) and shows that the vibrational frequencies are close to that for limonene in the gas and liquid phases.

The infrared spectra acquired to characterize the kinetics of the adsorption and desorption of limonene on SiO_2_ surface as a function of time are shown in Fig. 1b. Time dependent spectra display a steady increase in the spectral intensity of the C–H stretches upon adsorption of limonene on to the surface followed by a decrease and complete removal of C–H absorption bands due to limonene upon desorption, suggesting that limonene is adsorbed on the SiO_2_ surface *via* a reversible process. Furthermore, we calculated the surface concentration by combining volumetric measurements with C–H absorption band peak intensities for the adsorbed species as previously described (see Section S1†).^20^,^35^ Fig. 1c shows time course measurements of limonene concentrations on SiO_2_. Limonene was introduced to the system at *t* = 0 s, with increasing coverage observed until the adsorption of limonene on the surface was in equilibrium with the gas phase (*t* > 600 s). Desorption was then immediately initiated, and from that point onward, the surface coverage of limonene decreased with time. Additional IR studies along with modeling of kinetics of limonene on SiO_2_ surfaces are discussed after the next section.

### Limonene adsorption on SiO_2_ surfaces: MD simulations

#### Surface accommodation coefficient

The surface accommodation coefficient of limonene on the SiO_2_ surface was estimated by running MD trajectories of 100 ps duration that were initiated with the limonene center-of-mass velocity vector directed towards the SiO_2_ surface. This procedure was repeated for 100 randomly positioned and oriented limonene molecules at ∼2.5 nm above the SiO_2_ surface. Only 4 out of the 100 trajectories resulted in limonene scattered by the surface. Therefore, the surface accommodation coefficient of limonene on the SiO_2_ surface is estimated to be high, 0.96.

#### Structural characterization of limonene on the SiO_2_ surface

**Fig. 2:**
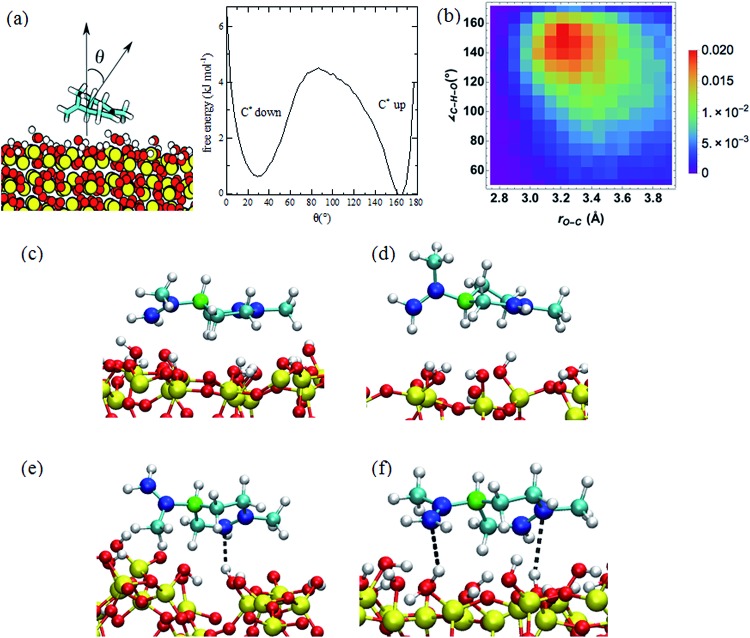
(a) Limonene reorientation free energy profile on the SiO_2_ surface computed from a 0.5 μs MD simulation. (b) Probability distribution of the O–C_sp^2^_ distance and C···H–O angle for the nearest hydrogen atoms of SiO_2_ to the sp^2^ carbon atoms. The probabilities are computed by dividing the count on each bin by the number of steps in the trajectory. (c and d) Snapshots of the limonene molecule in the C* up (c) and C* down (d) orientations. The chiral carbon atoms are colored green, the sp^2^ carbon atoms blue, the sp^3^ atoms cyan, the silicon atoms yellow, the oxygen atoms red, and the hydrogen atoms white. (e and f) Snapshots of structures corresponding to the one (e) and two (f) hydrogen-bonding interactions between the limonene and SiO_2_ surface. The dashed line depicts the hydrogen-bonding interaction.

During a 0.5 μs MD simulation, we observed that limonene has two predominant configurations, in which the six-membered ring is in contact with and parallel to the SiO_2_ surface. The limonene molecule stays in the more stable half-chair conformation with the propenyl group in the equatorial position during the entire trajectory.^36^ The two predominant configurations of the limonene molecule on the SiO_2_ surface are related by a roughly 180° rotation about the long axis and can be classified by the position of the chiral carbon atom, which we label C*. In one configuration, which we refer to as “C* up”, the C* atom is further from the SiO_2_ surface than in the other configuration, which we refer to as “C* down” (see Fig. 2c and d). Additionally, the populations of the C* up and C* down conformations are 43% and 35%, respectively. In the C* up configuration, the limonene molecule forms a more favorable interaction with the surface, namely, closer contact between the propenyl group and the SiO_2_ surface, which results in C* up orientation being the more probable conformation.

The free energy profile for the reorientation of the limonene molecule on the surface, *i.e.*, the transition from the C* up configuration to the C* down configuration, was calculated from the probability distribution of the angle, *θ*, between normal vectors in the limonene ring and the SiO_2_ surface (defined in Fig. 2a) according to (eqn (1)):

1 *F*(*θ*) = –*k*_B_*T* ln *p*(*θ*).

Here *p*(*θ*) is the probability distribution of *θ* acquired from the 0.5 μs MD simulation, *k*_B_ is Boltzmann's constant, and *T* is the temperature (295 K). The zero of the free energy profile was taken to be that of the most probable C* up configuration (*θ* ∼ 165°). According to the resulting free energy profile plotted in Fig. 2a, the free energy barrier to the transition from the C* down configuration to the C* up configuration is ∼4.0 kJ mol^–1^, and the barrier to the transition from the C* up configuration to the C* down configuration is ∼4.6 kJ mol^–1^.

The enthalpic barrier for the transition between the C* down and C* up configurations were estimated using the relative energies of the limonene–silica complex in the C* down, C* up, and vertical configurations of limonene with respect to the SiO_2_ cluster. The energies were calculated with M06-2X/6-311++G(d,p)//M06-2X/6-311G(d) electronic structure method.^30^ Results are summarized in Table 1 after correcting for basis set superposition error (BSSE).^31^ According to these calculations, the enthalpic barrier for passing from the C* up to the C* down configuration is 29.3 kJ mol^–1^.

**Table 1:** Relative energies of limonene–silica complex for different orientations of limonene molecule on SiO_2_ surface. Energies are reported relative to the lowest energy C* up configuration

Limonene orientation	Relative energy (kJ mol^–1^)
C* up	0.0
C* down	4.7
Vertical	29.3

The radial distribution functions (RDFs) between the hydrogen atoms of the SiO_2_ surface and the sp^3^ and sp^2^ carbon atoms of limonene are presented in Fig. S1.† The value of *r*_H–C_ at the first peak in the RDF is the distance at which preferred interactions occur. For the sp^3^ carbon atoms, the first peak in the RDF is at ∼3.5 Å, whereas for the sp^2^ atoms it is at ∼2.5 Å. The latter is indicative of a π–H bonding interaction between the hydrogen atoms of the SiO_2_ surface and the double bonds of the limonene molecule. The height and location of the first peak in the RDFs for the C* up and C* down configurations are the same, indicating that the strength of π–H bonding in the two configurations is similar. These values are in close agreement with the RDFs calculated using *ab initio* MD simulations.^37^

The histogram of the O–C_sp^2^_ distance and OH···C_sp^2^_ angle plotted in Fig. 2b demonstrates that the most probable O–C_sp^2^_ distance and OH···C_sp^2^_ angle occur at 3.2 Å and 155°, respectively, as expected for a hydrogen-bonding interaction. Based on Fig. 2b, we defined the criterion for π–H bonding as the O–C_sp^2^_ distance < 3.4 Å and the OH···C_sp^2^_ angle between 135° and 165°. According to this criterion, the probability of limonene engaging in one and two hydrogen bonds with the SiO_2_ surface is reported in the Table 2. Snapshots depicting the one and two hydrogen-bonding interactions between limonene and the SiO_2_ surface are depicted in Fig. 2e and f.

**Table 2:** The probability of formation of no, one, and two hydrogen-bonding interactions between the limonene molecule and SiO_2_ surface for the C* down, C* up configurations

Configuration	No HB	1 HB	2 HB
C* down	0.679	0.299	0.022
C* up	0.684	0.289	0.027

According to the results provided in Table 2, in the C* up configuration the probability of formation of two hydrogen-bonds between the limonene molecule and SiO_2_ increases by 0.5%, while the number of one hydrogen-bonding interactions decreases by 1.0% relative to the C* down configuration. As the propenyl group possesses more rotational flexibility compared to the rest of molecule, the probability of the sp^2^ C atoms in the propenyl group being involved in the hydrogen-bonding interaction with the surface is 8% lower than the sp^2^ carbon atoms located in the ring. For instance, 58% of 1 HB structures in the C* up configuration originate from the contacts between the double bond on the ring and surface.

#### Energetics of the desorption process

**Fig. 3:**
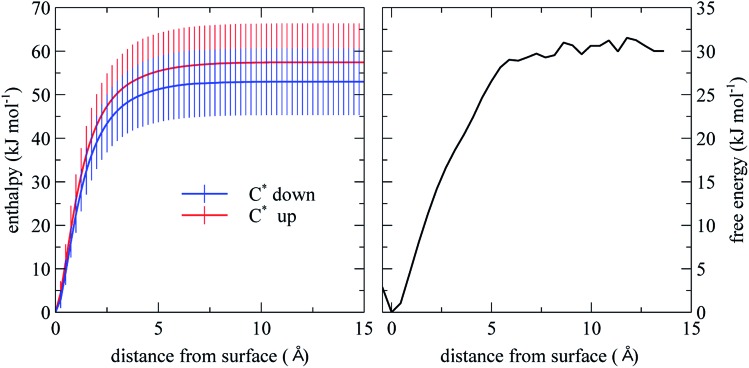
Desorption enthalpy and free energy computed from MD simulation. The error bars are based on the standard deviation of the energy computed for the 100 initial structures.

From the PMF for limonene desorption from the SiO_2_ surface plotted in Fig. 3 (right panel), we estimate that the desorption free energy is ∼30 kJ mol^–1^. We note that this value is a population-weighted average of both the C* down and C* up configurations. Using energy minimization, we estimated the enthalpy as a function of the distance between the limonene molecule and the SiO_2_ surface, separately for the two predominant limonene configurations, obtaining a desorption enthalpy of 57.0 ± 8.9 kJ mol^–1^ for the C* up configuration and 53 ± 7.6 kJ mol^–1^ for the C* down configuration (Fig. 3). However, considering the large error bars, the difference between the adsorption enthalpies of the two configurations is not statistically significant. The desorption enthalpy and free energy for limonene presented in this work is ∼10 kJ mol^–1^ larger than that of *α*-pinene reported by Geiger *et al.*, which is partly due to the extra C=C bond and more planar structure of limonene compared to *α*-pinene.^38^

### Adsorption and desorption kinetics as a function of pressure and temperature: transmission FTIR and kinetic modeling

**Fig. 4:**
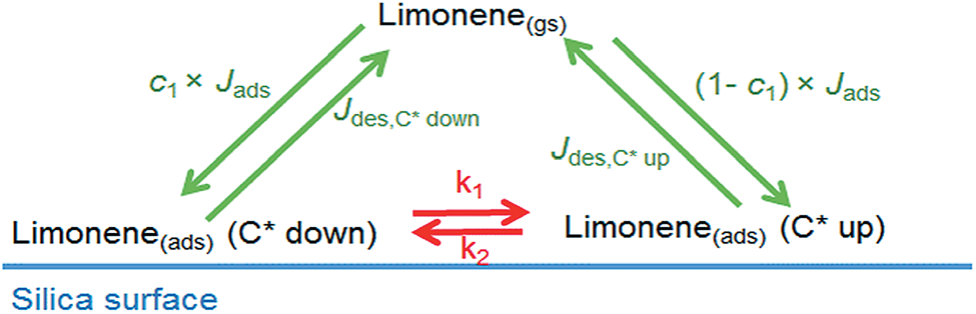
Schematic of the K2-SURF model for adsorption and desorption of limonene to a SiO_2_ surface. The subscripts ‘ads’ and ‘gs’ represent adsorbed molecules and near surface gas-phase molecules, respectively. *J*_ads_ is the adsorption flux which is equal to *α*_s,0,lim_ × *W*/4 × (1 – *θ*) × [Lim_(gs)_] while *J*_des_ is desorption flux which is equal to (1/*τ*_d,lim_) × [Lim_(ads)_]. *W* is the mean thermal velocity and *θ* is the surface coverage. A description of other parameters can be found in Table 3.

Vibrational spectroscopy is used to follow the kinetics of limonene adsorption/desorption on hydroxylated SiO_2_ at several pressures and temperature. The K2-SURF model was used to reproduce those experimental measurements. A simple schematic of the model is shown in Fig. 4. Table 3 summarizes the parameters used in K2-SURF to reproduce the experimental data. These parameters are based on MD simulations: the surface mass accommodation of limonene on a SiO_2_ surface should be close to 1 and the activation energies for the conversion of the C* up to C* down and C* down to C* up limonene configurations should be ∼29.3 kJ mol^–1^ and ∼24.6 kJ mol^–1^, respectively. Desorption lifetimes of limonene in K2-SURF needed to be set to 23–26 μs in order to reproduce the high limonene surface concentrations that were measured during the experiments. If these desorption lifetimes are inputted into an Arrhenius equation and a pre-exponential factor (*A*) of 1 × 10^14^ s^–1^ is assumed, an adsorption enthalpy (Δ*H*_ads_) of ∼53 kJ mol^–1^ is calculated. This is in very good agreement with the Δ*H*_ads_ value reported by MD simulations of 57.0 ± 8.9 kJ mol^–1^. The effective molecular cross-section of a limonene molecule was estimated as 0.55–0.79 nm^2^ by fitting to the experimental data, which is a reasonable range of values considering the molar mass and density of limonene.

**Table 3:** Parameters used in the K2-SURF model

Parameter	Parameter description	Parameter value
*α*_s,0,lim_	Surface accommodation coefficient on an adsorbate-free surface	1
*τ*_d,lim_	Desorption lifetime of limonene on the surface	2.3 × 10^4^ ns (Fig. 5), 2.6 × 10^4^ ns (Fig. S2)^*a*^
*k*_1_	First-order rate coefficient for the conversion of the C* down limonene configuration to the C* up limonene configuration	4.6 × 10^9^ s^–1^^*b*^
*k*_2_	First-order rate coefficient for the conversion of the C* up limonene configuration to the C* down limonene configuration	6.8 × 10^8^ s^–1^^*c*^
*σ*_lim_	Effective adsorption cross-section of a limonene molecule	0.55 nm^2^ (Fig. 5), 0.79 nm^2^ (Fig. S2 and 6)
*c*_1_	Fraction of limonene adsorbed as C* down, (1 – *c*_1_) is the fraction adsorbed as C* up	0.5

^*a*^For simplicity, the same value is assumed for the C* down and C* up configurations. Also note that these values are for room temperature (∼296 K). At other temperatures the following equation is used: (1/*τ*_d,lim_) = exp(–6423 × (1/*T*) + 32.24).

^*b*^The activation energy associated with this rate coefficient is 24.6 kJ mol^–1^ assuming a pre-exponential factor of 1 × 10^14^ s^–1^.

^*c*^The activation energy associated with this rate coefficient is 29.3 kJ mol^–1^ assuming a pre-exponential factor of 1 × 10^14^ s^–1^.

Sensitivity studies were performed to investigate the impact of changing parameters on the model output and to see whether any parameters were co-dependent as discussed below. The value of *c*_1_, which is the fraction of limonene which adsorbs as the C* down limonene configuration, was set to 0.5 in the model but could be set to any value between 0 and 1 without affecting the model output. The same model output could be achieved if the value of *α* was decreased and the value of *τ*_d_ was increased by the same factor, indicating that *α* and *τ*_d_ are co-dependent. The model output was insensitive to the values of *k*_1_ and *k*_2_, unless *τ*_d_ for the C* down and C* up limonene configurations were set to different values.

**Fig. 5:**
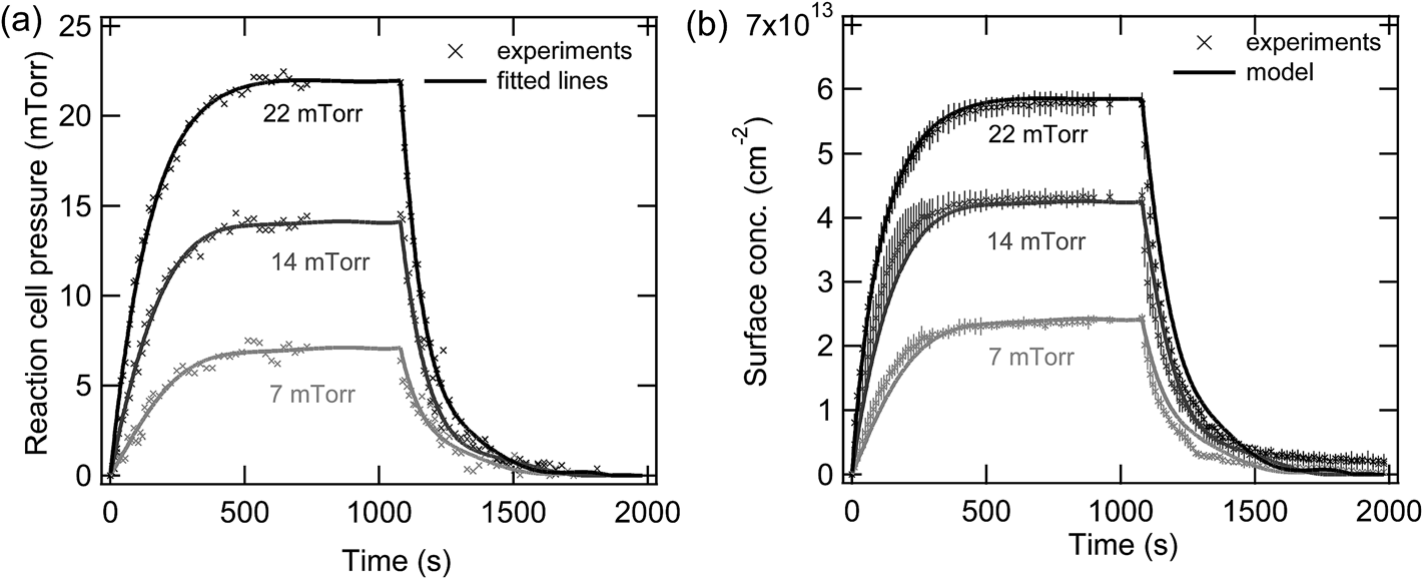
(a) Temporal evolution of limonene pressures in the reaction cell and (b) adsorbed limonene concentrations on SiO_2_ as a function of time for three different limonene equilibrium pressures, 7 (light grey), 14 (dark grey) and 22 (black) mTorr. Error bars represent the standard deviation between triplicate replicates of the same experiment.

Fig. 5 shows experimental measurements of the temporal evolution of the pressure in the reaction cell for three different equilibrium pressures. Individual polynomial lines for each of the three different experiments were used to fit these data sets and the evolution of the pressure as a function of time was then constrained to these fitted lines in the model. The data points in Fig. 5b shows experimental measurements of the temporal evolution of the adsorbed limonene concentration, while the lines are the model output. Table 4 summarizes the observed surface concentrations of limonene on SiO_2_ for various equilibrium pressures used in this study. The good agreement between the experimental measurements and the model suggest that the processes controlling the concentrations of limonene on the SiO_2_ surface are well understood as follows. The equilibrium between the gas-phase and surface-adsorbed limonene occurs on the order of microseconds, which is consistent with the short time scales of the parameters calculated by the MD simulations and shown in Table 3. The apparent slow adsorption and desorption of the limonene to the SiO_2_ surface, which occurs on the order of approximately 200 seconds, can be explained by slow gas-phase diffusion of the limonene into and out of the reaction cell, which leads to changing limonene pressures in the reaction cell over this time scale, as shown in Fig. 5a. Fig. S2† shows experimental measurements at different equilibrium pressures that were modelled using K2-SURF. Reaction cell pressures as a function of time were not available for these measurements and were therefore estimated and constrained in the model by fitting the three data sets shown in Fig. 5a using a single polynomial equation, which was normalized to the equilibrium pressure. Although the model can reproduce the measurements in Fig. S2† reasonably well, uncertainty in the reaction cell pressure is likely to be responsible for some of the deviations between the model and the measurements. It should also be noted that the parameters used when fitting the data in Fig. 5 and S2† were similar, as shown in Table 3.

**Table 4:** Calculated coverages of limonene on SiO_2_ for different pressures using volumetric measurements

Equilibrium pressure (mTorr)	Coverage (×10^13^ molecules/cm^2^)
6	2.4 ± 0.1
7	2.4 ± 0.1
8	2.7 ± 0.1
9	3.1 ± 0.2
10	3.2 ± 0.2
12	3.9 ± 0.2
13	4.1 ± 0.2
14	4.4 ± 0.2
16	4.3 ± 0.2
18	4.8 ± 0.2
21	5.7 ± 0.3
22	5.7 ± 0.3
23	5.8 ± 0.3
28	6.5 ± 0.3

**Fig. 6:**
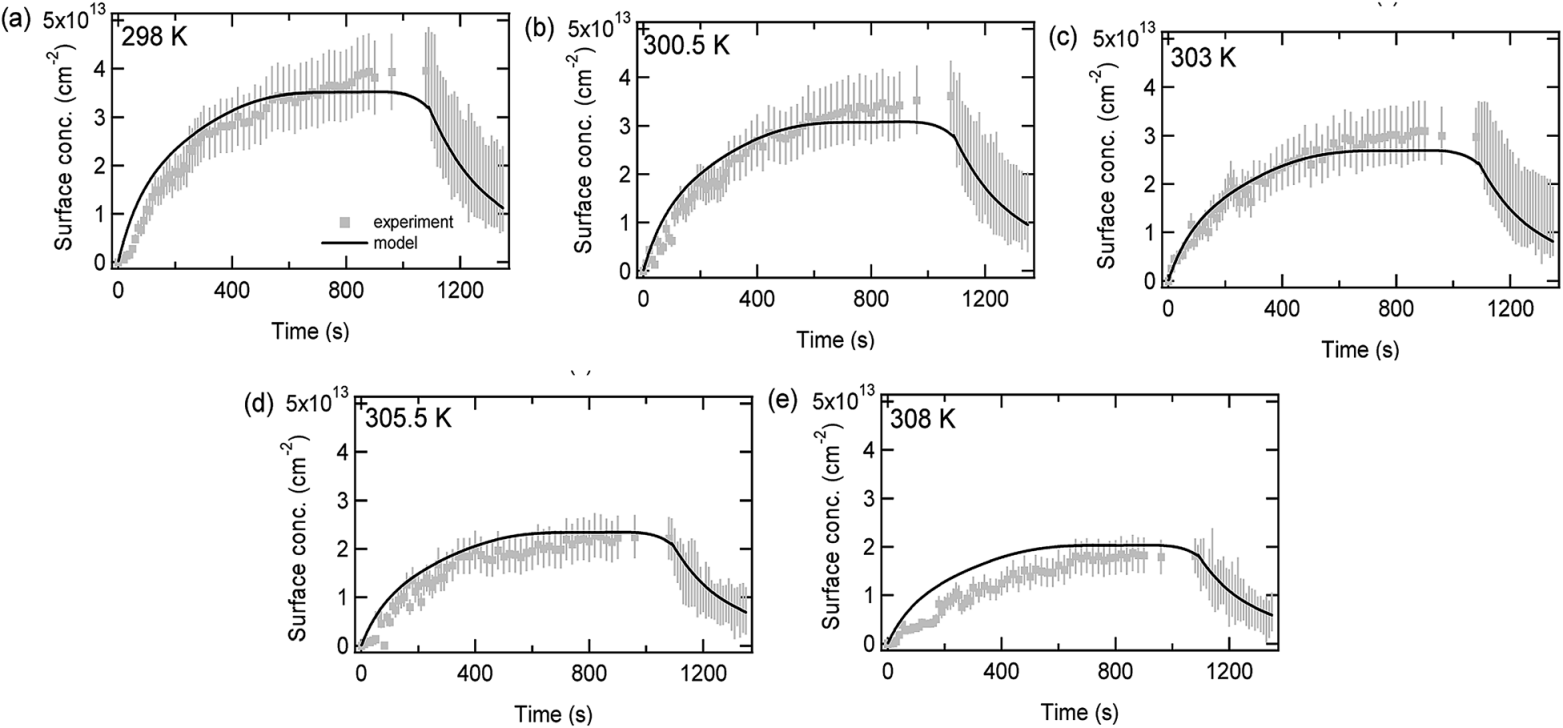
Adsorbed limonene concentrations on SiO_2_ as a function of time at an equilibrium pressure of 14 mTorr and at five different temperatures (a) 298 K, (b) 300.5 K, (c) 303 K, (d) 305.5 K, and (e) 308 K. A 20% error has been assumed for the adsorption part of the graphs while for the desorption part of the graphs a range of values are shown, representing experimental uncertainty described previously.

Fig. 6 compares experimental data (circles) and model predictions (lines) for limonene adsorption and desorption onto a SiO_2_ surface as a function of time for different temperatures. The decrease in the maximum adsorbed limonene concentration as the temperature increases can be explained by the first-order desorption rate coefficient following Arrhenius kinetics. The Arrhenius equation which was used to fit the data (1/*τ*_d,lim_ = exp(–6423 × (1/*T*) + 32.24)) had a pre-exponential factor (*A*) which was fixed to 1 × 10^14^ s^–1^, allowing the adsorption enthalpy (Δ*H*_ads_) to be calculated as –53.4 kJ mol^–1^. This is consistent with the pressure data, as when setting *A* to equal 1 × 10^14^ s^–1^ the value of Δ*H*_ads_ can be calculated as –(53.0–53.4 kJ mol^–1^). The values of Δ*H*_ads_ are larger than may be expected for purely physisorbed molecules, although this may be due to hydrogen bonding, and the values are lower than would be expected for strongly chemisorbed species.

### Conclusions and implications for indoor air chemistry

Classical MD simulation of limonene adsorption on SiO_2_ surface shows that the surface accommodation coefficient of limonene on SiO_2_ is close to 1. The limonene ring predominantly stays parallel to the SiO_2_ surface and involves in π–hydrogen bonding interaction with the hydroxyl groups of the surface. The adsorption enthalpy and Gibbs free energy were calculated to be –55 kJ mol^–1^ and –30 kJ mol^–1^, respectively. The kinetics of the limonene adsorption/desorption process have been investigated using a combination of surface experimental measurements obtained from vibrational spectroscopy and kinetic modeling with thermodynamic parameters obtained from MD simulations. The K2-SURF model and the proposed mechanism, inspired by the details of the adsorption process afforded by the MD simulations, are in good agreement with experimental coverage results obtained from vibrational spectroscopy.

Overall, this molecular level approach allows for the tracking of the dynamics of an organic film formation. Even in the absence of oxidants and high relative humidity (RH), these results should be applicable to isolated and quick activities when emissions containing limonene are released through products use and can then interaction with glass surfaces directly. Limonene adsorption occurs on very fast time scales, within microseconds, depending on the gas-phase concentrations. It must also be noted that SiO_2_ surface at ∼0% RH has a slightly higher number of free silanol groups compared to the surface at ∼50% RH, which suggests that our adsorption experiments on SiO_2_ in dry conditions (<1% RH) should be relevant for indoor relevant RH values.^39^ Furthermore, the surface dynamics will be different for a cold *versus* warm room since less limonene is adsorbed at higher temperatures. Importantly, the method developed for studying limonene adsorption/desorption on SiO_2_ surfaces can be further applied to other relevant indoor organic vapors and surfaces and, in the future, extended to understand the reaction chemistry of limonene on SiO_2_ surfaces in the presence of oxidants and water. This study thus represents an important step in understanding molecular details of indoor surface chemistry. These data and analyses can be used in future indoor air chemistry models which inform our understanding of indoor air quality.

## Conflicts of interest

There are no conflicts of interest.

## Supplementary Material

Supplementary information

Infographic
