# Supplementary material for: A molecular picture of surface interactions of organic compounds on prevalent indoor surfaces: limonene adsorption on SiO2
Source: Chem Sci. 2019 Jan 9;10(10):2906–14. doi: 10.1039/c8sc05560b (PMC6428143; doi:10.1039/c8sc05560b)
Supplement: Supplementary file 1 [file SC-010-C8SC05560B-s001.pdf]

Electronic Supplementary Material (ESI) for Chemical Science.
This journal is © The Royal Society of Chemistry 2019

Electronic Supplementary Informmation for

A Molecular Picture of Surface Interactions of Organic Compounds on Prevalent Indoor Surfaces: Limonene Adsorption on SiO_2

Yuan Fang[1], Pascale Lakey[2], Saleh Riahi[2], Andrew T. McDonald[1], Mona Shrestha[1], Douglas J. Tobias[2], Manabu Shiraiwa[2*], Vicki H. Grassian[1,3*]*

[1]Department of Chemistry & Biochemistry, University of California, San Diego, La Jolla, 92093, CA, USA

[2]Department of Chemistry, University of California, Irvine, 92697, CA, USA

[3]Scripps Institution of Oceanography & Department of Nanoengineering, University of California, San Diego, La Jolla, 92093, CA, USA

**Correspondence: Vicki H. Grassian (vhgrassian@ucsd.edu), Manabu Shiraiwa (m.shiraiwa@uci.edu) and Douglas J. Tobias (dtobias@uci.edu)*

Table S1. Vibrational mode assignment for limonene adsorbed on SiO_2 particle surfaces.

Mode assignment	SiO_2	Literature
ν_s(CH, sp^2)	3074, 3010 (ring)	3084, 3015[1]
ν(CH, sp^3)	2834, 2860, 2922, 2967	2836 – 2966[2]
Overtone		1780[3]
ν(C=C, alkene)	1645	1645[1,3,4]
δ(CH_2, CH_3)	1380, 1439, 1451	1377, 1453[3]

S1.*Surface concentration determination.* The amount of limonene adsorbed on silica surface is determined by volumetric measurements, as described in previous studies.[7,8] Equations (E1) to (E3) allow for calculation of the pressure represents the pressure calculation corresponding to the amount of adsorption which occurred on the silica surface. The amount of adsorption on the walls of the sample cell and mixing chamber, represented by the change of pressure in blank measurements (E1), has been subtracted from the amount adsorbed on the walls of the system as well as the sample loading(represented by the change in pressure during experiments (E2):

$$P_{blank,\,i} - P_{blank,\,eq} = \Delta P_{blank} \qquad \text{(E1)}$$

$$P_{exp,\,i} - P_{exp,\,eq} = \Delta P_{exp} \qquad \text{(E2)}$$

$$\Delta P = \Delta P_{exp} - \Delta P_{blank} \qquad \text{(E3)}$$

$P_{blank,\,i}$ and $P_{blank,\,eq}$ represent the initial pressure and equilibrium pressure of blank measurements, respectively. In the same manner, $P_{exp,\,i}$ and $P_{exp,\,eq}$ represent the pressures, but for experimental

measurements, and ΔP represents the change in pressure corresponding to the amount adsorbed by the sample only. By applying the ideal gas equation, this change in pressure can be converted to the number of molecules adsorbed on the surface. Note that this calibration was only done for a 100 mTorr initial introduction pressure. A conversion factor (in terms of molecules) is then applied by dividing the number of molecules adsorbed on the surface by the integrated absorbance peak area of the vibrational modes (from 2785 to 3115 cm^{-1}) using the spectrum collected at equilibrium (with the gas-phase contribution subtracted). This is subsequently used to determine the temporal evolution of the number of molecules adsorbed on the surface for other pressures, according to the integrated peak area which has been corrected for gas-phase diffusion for the same vibrational modes. Surface coverages were obtained by dividing the number of molecules adsorbed by the total surface area of the sample (BET surface area × mass of sample).

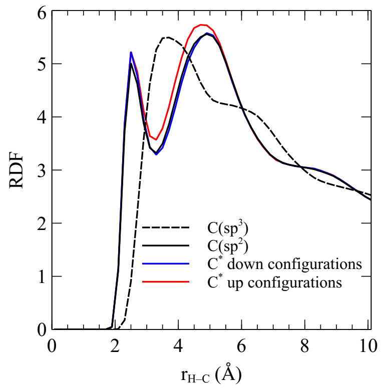

Figure S1. $H_{(silica)}$–C radial distribution functions computed for the sp^3 and sp^2 carbon atoms calculated for the full trajectory and structures corresponding to the C* up and C* down conformations.

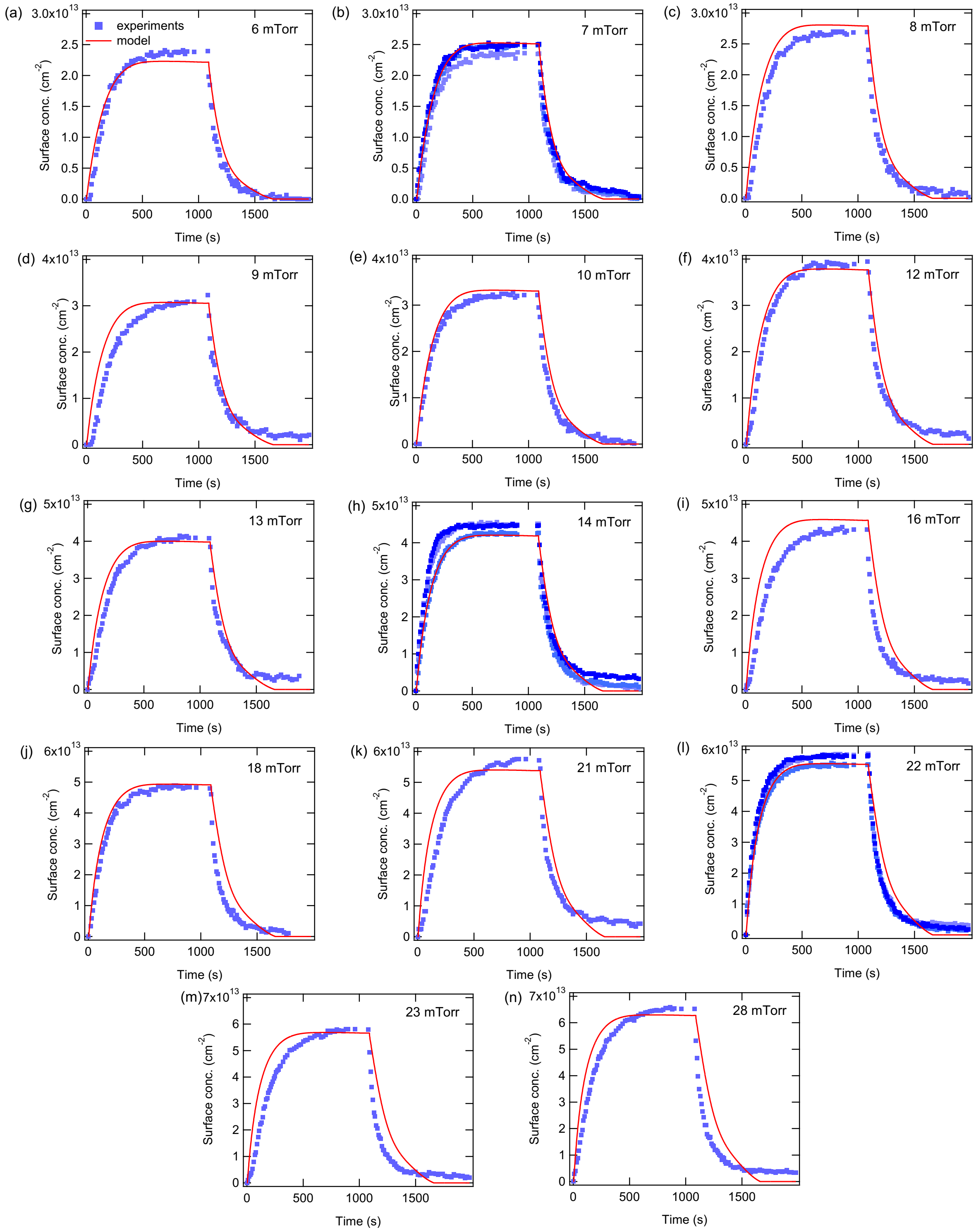

Figure S2: Adsorbed limonene concentrations on silica as a function of time for fourteen different limonene equilibrium pressures.

References

(1) (+)-Limonene in the Liquid Phase Using Vibrational Spectroscopy (IR, Raman, and VCD) and DFT Calculations. *Tetrahedron Asymmetry* **2009**, *20* (1), 89–97.

(2) Lederer, M. R.; Staniec, A. R.; Coates Fuentes, Z. L.; Van Ry, D. A.; Hinrichs, R. Z. Heterogeneous Reactions of Limonene on Mineral Dust: Impacts of Adsorbed Water and Nitric Acid. *J. Phys. Chem. A* **2016**, *120* (48), 9545–9556.

(3) O'Connor, R. T.; Goldblatt, L. A. Correlation of Ultraviolet and Infrared Spectra of Terpene Hydrocarbons. *Anal. Chem.* **1954**, *26* (11), 1726–1737.

(4) Subramanian, A.; Rodriguez-Saona, L. Chapter 7 - Fourier Transform Infrared (FTIR) Spectroscopy; Sun, D.-W. B. T.-I. S. for F. Q. A. and C., Ed.; Academic Press: San Diego, 2009; pp 145–178.

(5) Plath, K. L.; Axson, J. L.; Nelson, G. C.; Takahashi, K.; Skodje, R. T.; Vaidaa, V. Gas-Phase Vibrational Spectra of Glyoxylic Acid and Its Gem Diol Monohydrate. Implications for Atmospheric Chemistry. *React. Kinet. Catal. Lett.* **2009**, *96* (2), 209–224.

(6) Chapman, D. M.; Hester, R. E. Ab Initio Conformational Analysis of Trans-Cyclohexene. *The Journal of Physical Chemistry A* **1997**, *101*(18), 3382–3387.

(7) Goodman, A.L., Bernard, E.T., and Grassian, V.H. (2001). Spectroscopic study of nitric acid and water adsorption on oxide particles: Enhanced nitric acid uptake kinetics in the presence of adsorbed water. J. Phys. Chem. A *105*, 6443–6457.

(8) Tang, M., Larish, W.A., Fang, Y., Gankanda, A., and Grassian, V.H. (2016). Heterogeneous Reactions of Acetic Acid with Oxide Surfaces: Effects of Mineralogy and Relative Humidity. J. Phys. Chem. A *120*, 5609–5616.
