# Supplementary material for: A molecular picture of surface interactions of organic compounds on prevalent indoor surfaces: limonene adsorption on SiO2
Source: Chem Sci. 2019 Jan 9;10(10):2906–14. doi: 10.1039/c8sc05560b (PMC6428143; doi:10.1039/c8sc05560b)

Electronic Supplementary Material (ESI) for Chemical Science.
This journal is © The Royal Society of Chemistry 2019

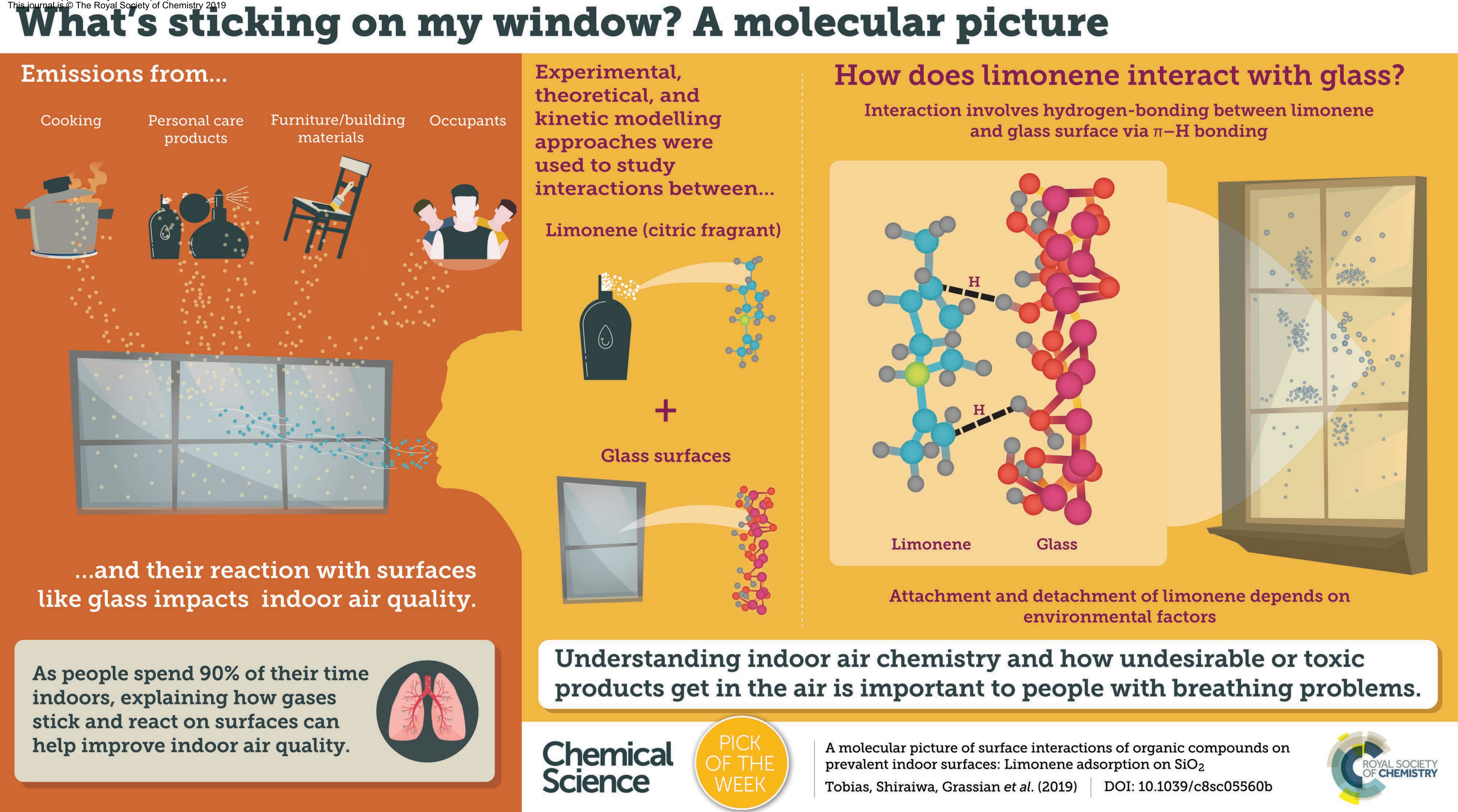

Supplement: Supplementary file 2 [file SC-010-C8SC05560B-s002.pdf]
